# Studying the Mechanisms of Developmental Vocal Learning and Adult Vocal Performance in Zebra Finches through Lentiviral Injection

**DOI:** 10.21769/BioProtoc.3006

**Published:** 2017-09-05

**Authors:** Zhimin Shi, Ofer Tchernichovski, XiaoChing Li

**Affiliations:** 1 Neuroscience Center of Excellence, LSU School of Medicine, New Orleans, LA, USA; 2 Department of Psychology, Hunter College, New York, NY, USA

**Keywords:** Zebra finch, Area X, miR-9, Lentivirus, Song learning, Song performance

## Abstract

Here we provide a detailed step-by-step protocol for using lentivirus to manipulate miRNA expression in Area X of juvenile zebra finches and for analyzing the consequences on song learning and song performance. This protocol has four parts: 1) making the lentiviral construct to overexpress miRNA miR-9; 2) packaging the lentiviral vector; 3) stereotaxic injection of the lentivirus into Area X of juvenile zebra finches; 4) analysis of song learning and song performance in juvenile and adult zebra finches. These methods complement the methods employed in recent works that showed changing *FoxP2* gene expression in Area X with lentivirus or adeno-associated virus leads to impairments in song behavior.

## [Background]

The zebra finch, with its well-characterized song behavior and the underlying neural circuitry, provides a unique animal model to study neural mechanisms underlying vocal communication and related sensory-motor learning. In recent years, several laboratories began using viral vectors to manipulate gene expression in the zebra finch brain and to study the functional consequences. These efforts are best illustrated by studies of the *FoxP2* gene, which encodes the forkhead box p2 transcription factor. The FoxP2 protein controls the expression of hundreds of downstream genes that have important roles in nervous system development. Mutations in the human *FoxP2* gene cause speech and language impairments (Lai et al., 2001). In songbirds, knockdown or overexpression of the *FoxP2* gene in Area X of zebra finches, a basal ganglia nucleus critical for vocal learning, profoundly impairs song behavior (Haesler et al., 2007; Murugan et al., 2013; Heston and White, 2015). These studies significantly extended the usage of the zebra finch model to study gene functions in neural circuit development, vocal communication behavior, as well as in speech and language-related neural developmental disorders. We recently reported that overexpression of miRNA miR-9 in Area X of juvenile zebra finches impairs song learning and performance (Shi et al., 2018). Hoping others might benefit from this study, here we provide step-by-step protocols for lentivirus cloning and production, stereotaxic injection of the virus into Area X of juveniles, and analysis of the impact of miR-9 overexpression on song learning and performance using the software Sound Analysis Pro (Tchernichovski et al., 2000). With minor modifications, these methods can be tailored to study other miRNAs or genes in vocal learning and performance in songbirds.

## Materials and Reagents

1. Pipette tips and Eppendorf tubes
2. 10 cm cell culture plates (Corning, catalog number: 430167)
3. 24-well cell culture plates (Corning, Costar^®^, catalog number: 3524)
4. 0.45 μm filter (Merck, catalog number: SCHVU01RE)
5. 30 ml Polyallomer conical centrifuge tube (Beckman Coulter, catalog number: 358126)
6. Insulin syringe (Smiths Medical, catalog number: 4429-1)
7. 25 G syringe needles
8. Betadine Surgical Scrub (Purdue Products)
9. Zebra finch tissue (*e.g.*, the brain)
10. XL 10 Gold Ultracompetent cells (Agilent Technologies, catalog number: 200314)
11. Oneshot Stbl3 competent *E. Coli* (Thermo Fisher Scientific, Invitrogen^™^, catalog number: C737303)
12. 293LTV Cells (Cell Biolabs, catalog number: LTV-100)
13. A lentiviral vector that contains the human ubiquitin promoter driving the expression of the mCherry fluorescent marker (Edbauer et al., 2010)
14. Lentivirus packaging plasmids psPAX2 and VSVG (Addgene, catalog numbers: 12260 and 35616)
15. PCR primers for miR-9 precursor amplification (Integrated DNA Technologies)
  Forward primer: 5’-GATGCTAGC TGTGTGTGTGGTTCCCGGTGGCAGCT-3’
  Reverse primer: 5’-CATGGCGCGCC GGACCCGCAGCCCTTACCTGGAGCCC-3’
  Note: The forward primer contains a NheI site and the reverse primer contains an AscI site (underlined).
16. PfuUltrall Fusion HS DNA polymerase (Agilent Technologies, catalog number: 600670)
17. Restriction enzymes Asci and Nhel (New England BioLabs, catalog numbers: R0558S, R0131S)
18. T4 DNA ligase (New England BioLabs, catalog number: M0202)
19. LB broth (Thermo Fisher Scientific, catalog number: 12780052)
20. Agar (Thermo Fisher Scientific, catalog number: 22700025)
21. Ampicillin (Sigma-Aldrich, catalog number: A0166-5G)
22. Genomic DNA isolation kit (QIAGEN, catalog number: 69504)
23. Gel extraction kit (QIAGEN, catalog number: 28704)
24. PCR purification kit (QIAGEN, catalog number: 28004)
25. EndoFree Plasmid Maxi Kit (QIAGEN, catalog number: 12362)
26. Agarose (Thermo Fisher Scientific, Invitrogen^™^, catalog number: 16500-500)
27. 50× TAE buffer (QIAGEN, catalog number: 129237)
28. IMDM Glutamax cell culture medium (Thermo Fisher Scientific, catalog number: 31980097)
29. Fetal bovine serum (FBS) (Thermo Fisher Scientific, catalog number: 10437028)
30. 2 M Calcium Solution
31. Penicillin-Streptomycin 5,000 U/ml (Thermo Fisher Scientific, catalog number: 15070063)
32. Cell culture medium IMDM supplemented with 10% FBS and 50 U/ml Penicillin-Streptomycin unless otherwise indicated
33. CalPhos Mammalian Transfection Kit (Takara Bio, catalog number: 631312)
34. Phosphate buffered saline (PBS, PH 7.4, Thermo Fisher Scientific, catalog number: 10010-023)
35. Ketamine (Henry Schein Ketathesia)
36. Xylazine (Henry Schein Vet)
37. Metacam (Boehringer Ingelheim Vetmedica)
38. Fluorescent dye (Thermo Fisher Scientific, catalog number: C34775)
39. Ethanol, 200 proof (Koptec)
40. Vetbond (3M)
41. Solution A (see Recipes)
42. Solution B (see Recipes)

## Equipment

1. Pipettes
2. Gel electrophoresis apparatus (Bio-Rad)
3. Water bath (37 °C and 42 °C, Precision)
4. Incubator with shaker (32 °C or 37 °C for growing bacteria)
5. Tissue culture hood
6. Tissue culture incubator temperature at 37 °C
7. Ultracentrifuge and SW28 rotor (Beckman Coulter, Optima, model: LE-80K)
8. Bench top centrifuge (Eppendorf, model: 5804 R)
9. Bench top centrifuge (Eppendorf, model: 5414 R)
10. ND-1000 Spectrophotometer (Thermo Fisher Scientific, model: NanoDrop^™^ 1000, catalog number: ND-1000)
11. Thermocycler (Bio-Rad)
12. Stereotaxic head holder (MyNeurolab)
13. Oil hydraulic micromanipulator (NARISHIGE, model: MO-10)
14. Glass needle puller (NARISHIGE, model: PC-10)
15. Glass Capillary (WIRETROL 1-5 μl) (Drummond Scientific, Wiretrol^®^, catalog number: 5-000-1001)
16. Track light (Motic, model: MLC-150C)
17. Thermal pat (Kent Scientific, model: DCT-15)
18. Scanning microscope with fluorescent light
19. Surgery tools: scissors and forceps (Fine Science Tools)
20. Microphone (Audio-Technica, catalog number: AT803b)
21. Amplifier (M-Audio, model: 2626)
22. Window Computer
23. Sound prove chamber (constructed following Sound Analysis Pro User Manual)
24. LED Light (Super Bright LEDs, catalog number: RLBN-NW30SMD)

## Software

1. Sound Analysis Program (SAP) Version 1.02 (Tchernichovski et al., 2000), http://soundanalysispro.com

## Procedure

Experiments involving lentivirus and animals should be approved by the Institutional Animal Care and Use Committee and the Institutional Biosafety Committee and follow institutional or national regulations. When working with bacteria or virus, all glassware, pipet tips, tubes, and solutions should be autoclaved when applicable before use. All surgery tools should be autoclaved before use, and surgical procedures should be performed under aseptic conditions.

**Figure 1.**
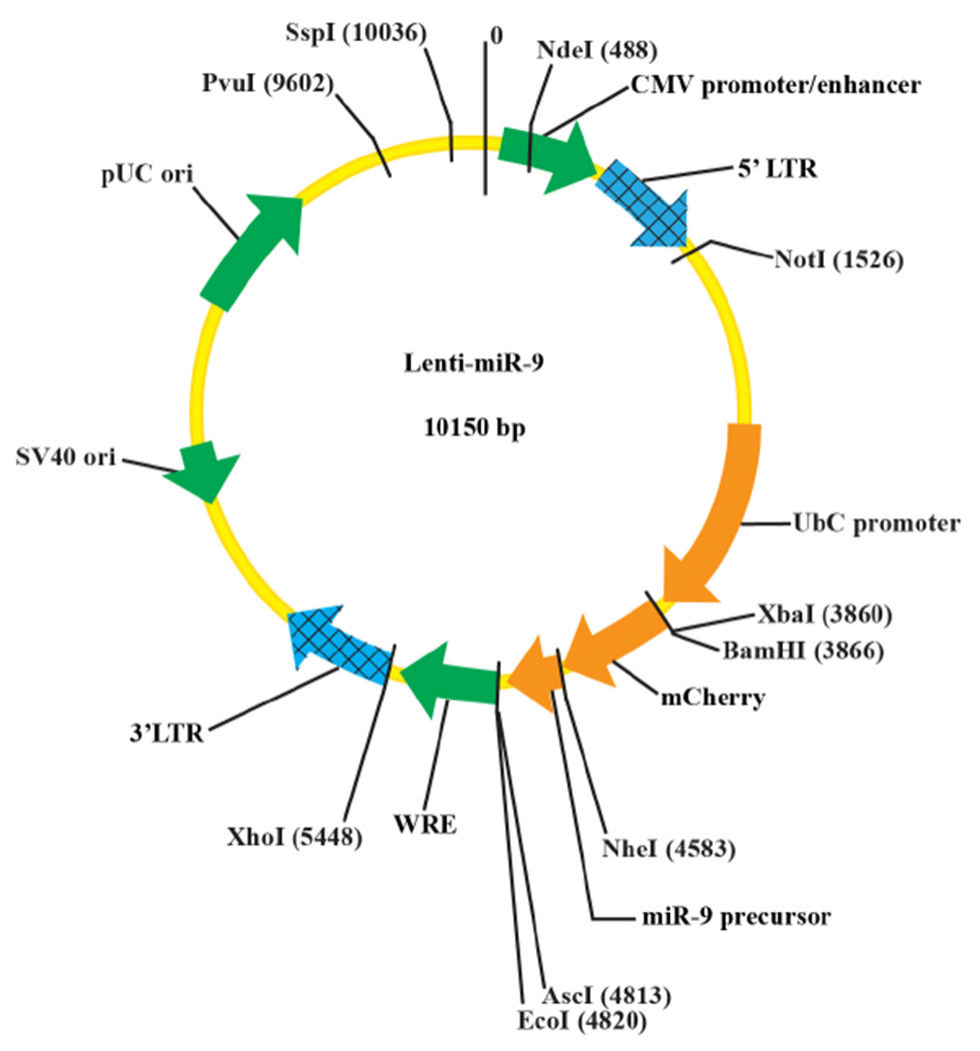
The plasmid map of the Lenti-miR-9 vector

- Clone the zebra finch miR-9 gene into a lentiviral vector
  1. Isolate genomic DNA from any zebra finch tissue (*e.g.*, the brain) using the QIAGEN genomic DNA isolation kit.
  2. Amplify the zebra finch miR-9 gene from the genomic DNA using PCR (denaturing: 95 °C/10 sec; annealing: 54 °C/25 sec; and extension: 72 °C/25 sec; 40 cycles).
  3. Separate the PCR product by electrophoresis on 1.5% agarose gel.
  4. Cut out the 290 bp band from the gel, and purify the DNA fragment using the QIAGEN Gel Extraction kit.
  5. Digest the PCR product with the restriction enzymes NheI and AscI at 37 °C for 2-3 h.
  6. Digest the lentiviral vector with restriction enzymes NheI and AscI at 37 °C for 2-3 h.
  7. Purify the digested lentiviral vector by gel electrophoresis followed by gel extraction.
  8. Ligate the miR-9 fragment to the lentiviral vector (molar ratio: 5:1) with T4 DNA ligase in 20 μl ligation buffer at 4 °C overnight.
  9. Transform the Stbl3 cells with DNA ligation mix.
  10. Plate the transformed Stbl3 cells onto LB agar plate with Ampicillin (100 mg/ml).
  11. Grow the bacteria at 32 °C for 20 h.
  12. Pick a single colony and grow in 250 ml LB broth/ampicillin at 32 °C for 15-20 h.
  13. Purify the plasmid DNA using the QIAGEN EndoFree Plasmid Maxi Kit.
  14. Re-suspend the plasmid DNA in 10 mM Tris buffer (pH 7.5).
  15. Quantify the plasmid DNA with Nanodrop.
  16. Validate the plasmid DNA by sequencing and/or digestion with restriction enzymes NheI and AscI (see the plasmid map in Figure 1).
  17. Prepare the packaging plasmids psPAX2 and VSVG similarly as described in Steps A9-A15, except XL10 Gold Ultracompetent cells are used and the bacteria are grown at 37 °C.
- Production of lentivirus
  1. Seed 293LTV cells 3 × 10^6^/10 cm plates (typically 6 plates) in IMDM medium, supplemented with antibiotics (unless otherwise indicated) and 10% FBS the day before transfection.
  2. Replace 75% of the medium with IMDM (no FBS) next day, 2 h before transfection (cells are about 70% confluent).
  3. Prepare solution A and solution B in separate tubes (see Recipes below).
  4. Add solution B to solution A dropwise while gently shake the transfection mix (A + B).
  5. Let the transfection mix sit at room temperature for 15 min.
  6. Gently add transfection mix dropwise to cell culture plate (1.4 ml transfection solution per 10 cm plate).
  7. Incubate the transfected cells at 37 °C for 8-10 h.
  8. Remove and discard the calcium phosphate-containing medium and replace with 8 ml IMDM containing 2% FBS.
  9. Collect the virus-containing cell culture medium at 48 h after transfection (store at 4 °C until Step B11) and replace the medium with 8 ml IMDM containing 2% FBS.
  10. Collect the virus-containing cell culture medium at 72 h after transfection (cells can be discarded afterward).
  11. Combine the collected cell culture medium and spin at 720 *× g* (2,000 rpm, Eppendorf centrifuge, 5804 R)/10 min at 4 °C.
  12. Save the supernatant and filter it with a sterile 0.45 μm filter.
  13. Spin the supernatant at 82,700 (r_av_) *× g* (25,000 rpm, ultracentrifuge) for 2 h at 4 °C.
  14. Discard supernatant and rinse the pellet briefly with PBS.
  15. Re-suspend the pellet in 50-60 μl PBS at 4 °C overnight.
  16. Bleach all waste medium and plastic wares before throwing them away.

### Titer the virus

17. Seed 293LTV cells in a 24-well plate to 2 × 10^4^ cells per well in IMDM w/10% FBS.
18. Twenty-four hours later, change medium to IMDM w/2% FBS.
19. Make serial viral dilutions with IMDM medium: 10^−1^, 10^−2^, 10^−3^, 10^−4^, 10^−5^, and 10^−6^.
20. Add 1 μl of each viral dilution to cells in each well, three wells per viral dilution.
21. Seventy-two hours later, count the number of fluorescent cells per well starting from the 10^−5^ or 10^−6^ dilution and average the cell counts from the triplicate wells.
22. The titer is the number of fluorescent cells per well times the dilution factor.
  *e.g.,* if the cell count is 3/well at 10^−6^ dilution, the titer would be 3 × 10^6^ IU.
  Typically we obtain a titer about 2-3 × 10^6^ IU/μl (IU = infection unit).

**Figure 2.**
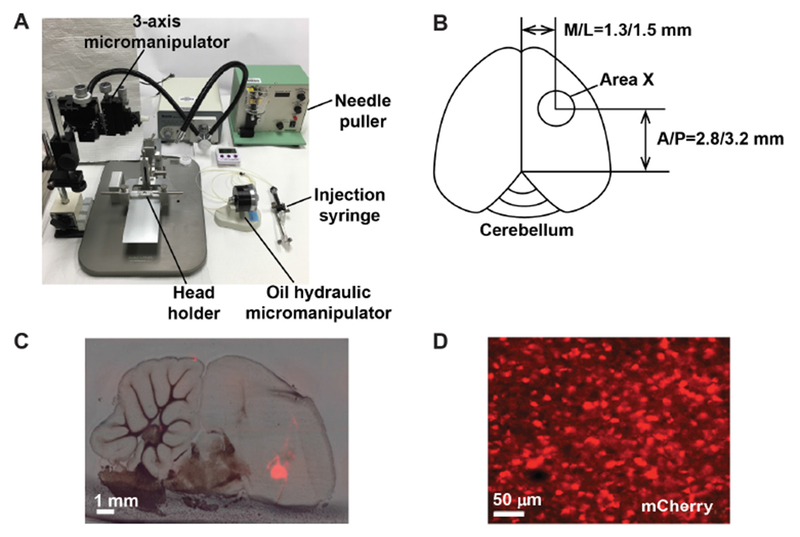
Injecting the lentivirus into Area X of the zebra finch brain. A. Stereotaxic setup for surgical procedures. B. Schematic illustration showing the coordinates for viral injection into Area X. C. Exemplar brain section showing virally expressed mCherry signal in Area X. D. mCherry-labeled neurons in Area X.

**Figure 3.**
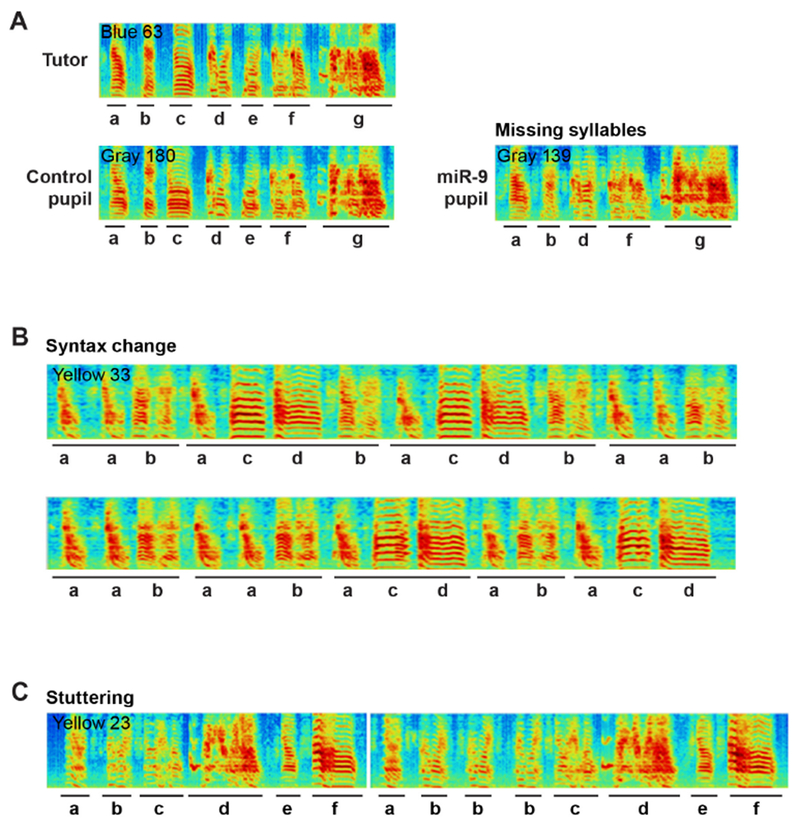
Representative sonograms of miR-9 pupils showing examples of A) Missing syllables, B) Changes in syllable sequence and C) Syllable stuttering. A. Image showing that the song motifs of both the tutor and the control pupil have 7 syllables, whereas the miR-9 pupil’s motif has 5 syllables and syllables c and e are missing. B. Image showing the scrambled syllable sequence of a miR-9 pupil. C. Image showing two motifs of a miR-9 pupil; syllable b is repeated three times in the second motif.

C. Injection of the lentivirus into juvenile Area X
  1. Prepare the male juvenile finches for injection by removing their father at day 10, and keeping them with their mothers in a sound attenuated chamber until day 30. Viral injection is performed at 25 ± 1 days of age.
  2. Weigh and anesthetize the animal by intramuscular injection with 24 μg/Ketamine-12 μg/Xylazine per g of body weight.
  3. Mount the animal onto the stereotaxic head holder platform with the tail up by 10 degree, and tighten the mouth bar and the ear bars.
  4. Disinfect the scalp with iodine and pluck the feather away from the top of head.
  5. Open the scalp along the middle line about 1-1.2 cm using a pair of scissors.
  6. Pull a glass injection needle using a needle puller. The heating temperature can be adjusted by turning the dial so that the inner diameter at the needle tip is 25-30 μm (can be done beforehand).
  7. Briefly spin the virus solution before injection for 5 min at 9,300 *× g* (10,000 rpm, Eppendorf centrifuge, 5414 R) at 4 °C.
  8. Fill the injection needle with 1 μl mineral oil, 1-2 μl viral solution, and 0.5 μl mineral oil.
  9. Install the injection needle onto the stereotaxic manipulator.
  10. Move the injection needle to the bregma point using the stereotaxic manipulator and record the anterior/posterior and medial/lateral coordinates (this is the reference point for the injection coordinates).
  11. Move the injection needle to above Area X (middle point of A/P and M/L injection coordinates) and make a mark.
  12. Open a small window 1-1.5 mm^2^ on the skull at the marked site using a 25 G syringe needle.
  13. Make an opening in the dura with a 25 G needle to facilitate entry of the glass needle.
  14. Inject each Area X at 6 or 8 sites at the following coordinates (Figure 2B): anterior/posterior, 2.8 and 3.2 mm; medial/lateral, 1.3 and 1.5 mm; dorsal/ventral, 4.2 and 4.4 mm (from the surface of the skull). For behavioral experiments, virus is injected bilaterally.
  15. Inject 120 nl viral solution at each site over a period of 2 min using the hydraulic pressure device.
  16. Let the injection needle remain at the site for 2 min before injection and 5 min after injection before removal to facilitate diffusion of viral solution.
  17. Put back the skull bone to the opening and close the skin (one side slightly over another side) and apply Vetbond to seal the scalp.
  18. Put the animal on a thermal pat at temperature 30 °C until it wakes up (takes about 30 min).
  19. Return the animal to the home cage.
  20. Disinfect the surgery area with 70% ethanol, bleach the injection needles and throw them into a sharp waste container, wash and autoclave surgical tools.
  21. Record the following information: injection date, animal ID, injection agents, coordinates and volume of injection.
D. Song recording and analysis
  1. Keep the injected juveniles with their mothers until day 30, give an adult male tutor to each injected juvenile, and keep the pair in a sound-attenuated chamber from day 30 to day 70.
  2. Record undirected songs for each juvenile pupil at specified age for two days in the absence of the tutor.
  3. Sort manually all song files recorded in one day from 8 AM to 12 PM and eliminate files representing cage noise (this step can be done automatically using the SAP).
  4. Select 20 song files approximately evenly spread across the entire set of sorted song files (*e.g.*, select the first, 11th, 21st, 31st, *etc.* if there are 200 song files) for each pupil.
  5. Counting the average number of syllables per motif
    Count manually the total number of syllables and the total number of motifs in 20 pupil song files (50-80 motif renditions) and 10 tutor song files (25-40 motif renditions). In cases when a pupil or a tutor song has multiple versions of motifs, include all versions in counting and exclude partial motifs typically appearing at the beginning or the end of a song file. Divide the total number of syllables by the total number of motifs for both the pupil and its tutor. Compare the number of syllables per motif for each pupil to that of its tutor.
  6. Counting the number of missing syllable
    Count manually the number of syllable types (A, B, C, D, *etc.*) in 20 pupil song files (50-80 motif renditions) and 10 tutor song files (25-40 motif renditions). If a syllable type occurs only in the tutor’s song, but not in the pupil’s song, or if the frequency of a syllable in a pupil’s song is less than 10% of its frequency in the tutor’s song, it is defined as a missing syllable.
  7. Motif similarity analysis
    Compare 20 pupil motifs with 10 tutor motifs and obtain a motif similarity score for each comparison using the default asymmetric time course mode of SAP. Average % similarity of the 200 pairwise comparisons to obtain a motif similarity score.
  8. Maximum motif similarity analysis
    Rank the 200 motif similarity measurements (20 pupil motifs × 10 tutor motifs) for each pupil and average the 10 highest values (top 5%) to obtain the maximum motif similarity score.
  9. Syllable accuracy analysis
    Measure the accuracy score for each syllable of a pupil’s song motif in 20 renditions using the default asymmetric mode of SAP. Average the accuracy scores of all syllables in a pupil’s motif to obtain a syllable accuracy score for that pupil.
  10. Syllable feature analysis
    Measure each syllable feature (duration, mean frequency, goodness of pitch, frequency modulation, and Wiener entropy) for each syllable in 20 pupil motif renditions and 10 tutor motif renditions using the SAP, and average the measurements for all renditions.
    Calculate the difference from the tutor (%) for each acoustic feature and for each syllable: (pupil’s measurement - tutor’s measurement)/tutor’s measurement.
    Average the percentage difference values of all syllables for each syllable feature.
  11. Syllable feature variation
    Calculate a coefficient of variation for each acoustic feature for 20 renditions of a syllable, and average the coefficients of variation for all syllable types for each acoustic feature.
  12. Syllable transition entropy analysis
    - Segment all songs recorded in two days from 8 AM to 12 PM using the auto-segmentation function of SAP (Typically 10,000-19,000 syllables can be obtained).
    - Classify these syllables into types (clusters) using the clustering module of SAP.
    - Visually validate the clusters by matching clusters with syllable types in the sonograms, and manually correct obvious cases of false classification (*e.g.,* due to segmentation inconsistency).
    - Calculate the transition frequencies between all pairs of syllable types, which results in a matrix. For example, for a song motif containing five syllable types (A, B, C, D, and E), calculate syllable transition frequencies for A to A, A to B, A to C, A to D, A to E; B to A, B to B, B to C, and so on.
    - For each syllable type t (each row in the matrix), calculate the relative transition probability: pt = transition frequency between a syllable pair divided by the sum of transition frequencies of all syllable pairs in a row.
    - Compute transition entropy for each syllable type t: Entropyt = sum [pt × log(pt)].
    - Compute a weighted transition entropy for each syllable type:
      Entropytw = Entropyt × syllable weight, so as to give higher weight to the more frequent syllable types. A syllable weight is defined as the transition frequencies of a given syllable type (sum of a row in the matrix) divided by the sum of transition frequencies of all syllable types (sum of the entire matrix).
    - Finally, calculate the overall transition entropy for a song by averaging transition entropies of all its syllable types.
  13. Quantifying the amount of singing
    Segment all song files recorded for each bird between 8 AM to 12 PM in two days using the batch mode of SAP. This process generates the total number of syllables a bird sings during the indicated time.
  14. Recording female-directed songs
    Record female-directed songs manually between 8:00-11:00 AM (A female-directed song is defined as a song that a male sings toward a female as observed by an experimenter). A male is induced to sing female-directed songs by presenting one or two females in a nearby cage. If needed, females can be changed every 10 min.
  15. Constant fundamental frequency analysis
    - Analyze the same set of syllables that contain a segment with a constant fundamental frequency (harmonic stacks), and that are produced in the contexts of both undirected singing and female-directed singing.
    - Measure the constant fundamental frequency using the SAP. Typically, include 20-40 syllable renditions from 20 song files in each context in the analysis.
    - Exemplar sonograms of pupils injected with the control or miR-9 virus are shown in Figure 3.
  16. Statistical analysis
    For song behavioral experiments, we typically use 6-8 animals per treatment group, and include 10-20 motif renditions per animal and multiple syllables per motif in the analysis as indicated above. We use various statistical analysis methods such as t-test, paired t-test, ANOVA and/or Mann-Whitney test to evaluate the data and use P < 0.05 as the cutoff for significance.

## Notes

1. Molecular cloning
  For standard molecular biology work, such as genomic DNA isolation, PCR product purification, plasmid DNA purification, restriction enzyme digestion and ligation, we follow the manufacturers’ instructions, especially when using QIAGEN kits.
2. Handling lentivirus
  Lentiviruses should not be frozen and thawed multiple cycles and/or stored for long periods of time, which could cause a drastic drop in viral titer. We routinely prepare fresh virus for each injection experiment. Thus, the injection time (depending on the age of the animals) and viral preparation need to be coordinated. Typically, viral preparation starting from growing cells takes about 10 days. Once made, the lentivirus can be kept at 4 °C for 3-4 days without a significant drop in titer.
3. Testing injection coordinates
  The injection coordinates can be tested by injecting a fluorescent dye into a targeted area. After injection, animals are killed, and brains are sectioned into 100 μm-thick sections. The sections are imaged with bright and fluorescent lights using a scanning scope (2× lens). Images are merged in Photoshop to check whether injection hits Area X.
4. Validating injection sites
  We recommend examining the injection sites after the last behavioral experiment. This can be done by sectioning the brains and imaging brain sections with both regular light and fluorescent light. The two sets of images are merged to examine whether injection sites are within Area X (Figure 2C). If the mCherry fluorescent signal is outside of Area X, the animal should be excluded from behavioral analysis or should be used as a control. The average area exhibiting strong mCherry signal typically accounts for ~20% of total Area X volume.
5. Tutors
  We typically use a heterogeneous group of adult male tutors to ensure that the observed impairment in song learning is not dependent on a specific tutor song. However, it is ideal that a subset of pupils injected with the experimental or the control virus are tutored by the same tutor. This will help ensure that any difference in song learning is not due to some tutor songs are more difficult to learn than others. Because tutor songs can change gradually, pupil songs should be compared to recently recorded tutor songs (within six months of tutoring).
6. Song analysis
  Because zebra finch songs exhibit daily structural oscillation (Deregnaucourt et al., 2005), we recommend consistently analyzing songs produced during a specified time period. We also recommend that two investigators validate the key experimental results with at least one blind to the treatment groups.

## Recipes

1. Solution A
  Add components in the following order:

**Table:** 

Lenti-vector plasmid DNA	67 μg
psPAX2	50 μg
VSVG	34 μg
2 M Calcium Solution	347.2 μl
Sterile H_2_O	to 2,800 μl
2. Solution B
  2,800 μl 2× HBS

## References

[R1] Deregnaucourt S, Mitra PP, Feher O, Pytte C and Tchernichovski O (2005). How sleep affects the developmental learning of bird song. Nature 433(7027): 710–716. 15716944 10.1038/nature03275

[R2] Edbauer D, Neilson JR, Foster KA, Wang CF, Seeburg DP, Batterton MN, Tada T, Dolan BM, Sharp PA and Sheng M (2010). Regulation of synaptic structure and function by FMRP-associated microRNAs miR-125b and miR-132. Neuron 65(3): 373–384. 20159450 10.1016/j.neuron.2010.01.005 PMC5018398

[R3] Haesler S, Rochefort C, Georgi B, Licznerski P, Osten P, and Scharff C (2007). Incomplete and inaccurate vocal imitation after knockdown of *FoxP2* in songbird basal ganglia nucleus Area X. PLoS Biol 5: e321. 18052609 10.1371/journal.pbio.0050321 PMC2100148

[R4] Heston JB and White SA (2015). Behavior-linked FoxP2 regulation enables zebra finch vocal learning. J Neurosci 35(7): 2885–2894. 25698728 10.1523/JNEUROSCI.3715-14.2015 PMC4331621

[R5] Lai CS, Fisher SE, Hurst JA, Vargha-Khadem F and Monaco AP (2001). A forkhead-domain gene is mutated in a severe speech and language disorder. Nature 413(6855): 519–523. 11586359 10.1038/35097076

[R6] Murugan M, Harward S, Scharff C and Mooney R (2013). Diminished FoxP2 levels affect dopaminergic modulation of corticostriatal signaling important to song variability. Neuron 80(6): 1464–1476. 24268418 10.1016/j.neuron.2013.09.021 PMC3881289

[R7] Shi Z, Piccus Z, Zhang X, Yang H, Jarrell H, Ding Y, Teng Z, Tchernichovski O and Li X (2018). miR-9 regulates basal ganglia-dependent developmental vocal learning and adult vocal performance in songbirds. Elife 7: e29087. 29345619 10.7554/eLife.29087 PMC5800847

[R8] Tchernichovski O, Nottebohm F, Ho CE, Pesaran B and Mitra PP (2000). A procedure for an automated measurement of song similarity. Anim Behav 59(6): 1167–1176. 10877896 10.1006/anbe.1999.1416

